# Gastroenteritis in middle-aged and elderly adults in rural China: associations with sociodemographic, lifestyle, and dietary factors

**DOI:** 10.1186/s12876-026-04916-0

**Published:** 2026-05-18

**Authors:** Yanli Gao, Da Pan, Ming Su, Yuanyuan Wang, Jingya Han, Xin Wang, Qingyang Yan, Junlei Xu, Zhiwen Zhang, Ligang Yang, Hui Xia, Wang Liao, Shaokang Wang, Guiju Sun

**Affiliations:** 1 Key Laboratory of Environmental Medicine and Engineering of Ministry of Education, and Department of Nutrition and Food Hygiene, School of Public Health, Southeast University, Nanjing, 210009 People’s Republic of China; 2 2Huai’an District Center for Disease Control and Prevention, Huai’an, 223200 People’s Republic of China; 3 School of Medicine, Xizang Minzu University, Xianyang, 712082 People’s Republic of China

**Keywords:** Sociodemographic characteristics, Lifestyle, Dietary behavior, Gastroenteritis, Middle-aged, Elderly population

## Abstract

**Background:**

Gastroenteritis remains a common gastrointestinal disorder with health and socioeconomic impacts. However, data on its epidemiological characteristics in rural areas of China are limited. This study aimed to investigate the factors associated with gastroenteritis among adults in a rural population in China.

**Methods:**

This cross-sectional study analyzed data from the Early Diagnosis and Early Treatment Project of Esophageal Cancer (2011–2017). A total of 11,518 participants aged 35–75 years completed face-to-face questionnaires. Univariate and multivariate logistic regression models estimated crude and adjusted odds ratios with 95% confidence intervals.

**Results:**

Of all participants, 2,849 were diagnosed with gastroenteritis. Increased risk was associated with female, increased BMI, annual income, fast eating, irregular diet, high blood pressure, salty diet, spicy diet, excessive smoking, exposure to secondhand smoke, and consumption of fatty meats, corn, cornmeal, pickled foods, and fried foods. Higher intake of vegetables, fruits, and animal liver was linked to reduced risk.

**Conclusions:**

Gastroenteritis in rural Huai'an is closely associated with tobacco use, unhealthy dietary habits, and environmental exposures. Public health interventions focusing on smoking cessation, dietary improvement, and environmental health promotion may provide support for its primary prevention in this population.

**Supplementary Information:**

The online version contains supplementary material available at 10.1186/s12876-026-04916-0.

## Introduction

Gastroenteritis is a common clinical inflammatory disease of the digestive system, whose main pathology is characterized by an inflammatory reaction of the gastrointestinal mucosa [1]. According to epidemiological data from the World Health Organization (WHO), the disease presents a significant disease burden globally, especially in areas with less developed healthcare. Etiologically, gastroenteritis can be divided into infectious (pathogens include bacteria, viruses, and parasites) and non-infectious (triggers include adverse drug reactions, toxin exposure, and autoimmune abnormalities) types, and its typical clinical symptoms include diarrhea, vomiting, abdominal pain, and fever [2].

In rural areas of China, the risk of gastroenteritis may be significantly higher among middle-aged and older adults due to a combination of factors such as imbalance in the allocation of healthcare resources, poor public health infrastructure, and the aging of the population. Existing epidemiological evidence suggests that the pathogenesis of gastroenteritis is multifactorial, involving complex interactions among sociodemographic factors (e.g., age, gender, education, and socioeconomic status) [3], lifestyle (including smoking, alcohol consumption, exercise habits, and sleep quality) [4], and dietary structure (e.g., dietary fiber intake, sodium intake, and food hygiene) [5]. However, there are still significant data gaps in the studies of rural Chinese middle-aged and elderly populations, especially the lack of systematic research on the dose–response relationship and potential effect modification between social and behavioral factors and the risk of gastroenteritis.

This study was designed as a population-based cross-sectional epidemiological study based on the “Early Diagnosis and Early Treatment Project of Esophageal Cancer (EDETPEC)”, a public health program initiated in Huai'an since 2010. The study aimed to explore in depth the associations between socio-demographic characteristics, lifestyle and dietary patterns and the risk of developing gastroenteritis, and to systematically analyze the interactions among the influencing factors. By revealing these potential associations, this study not only provides evidence-based support for the primary prevention of gastroenteritis in this region, but also offers a theoretical basis for developing targeted public health intervention strategies, which may help reduce the burden of gastroenteritis in the middle-aged and elderly population in rural China.

## Materials and methods

### Study population

Medical personnel from each village health office were tasked with contacting eligible target populations for subject recruitment, resulting in an overall compliance rate of approximately 80%. Between January 2011 and December 2017, the study team enrolled a total of 11,655 permanent residents aged 35 to 75 years (4,500 males and 7,155 females) as part of the EDETPEC program in Huai'an District, Huai'an City, Jiangsu Province, China. Following stringent exclusion criteria, individuals with any history of malignancy were excluded from the study. Ultimately, we identified 11,518 eligible subjects (4,431 males and 7,087 females), which included 2,849 cases of gastroenteritis alongside normal controls versus a total of 8,669 control subjects. The study adhered rigorously to the ethical guidelines set forth by the Declaration of Helsinki. Furthermore, the study protocol received review and approval from the Institutional Review Board at Zhongda Hospital affiliated with Southeast University (Jiangsu Province, China) under Approval No. 2012DllKY19.0; all participants provided written informed consent prior to their involvement in this research.

### Disease diagnosis

The diagnosis of gastroenteritis was established based on participants' self-reported history of gastrointestinal diseases during the study period, integrated with a diagnostic framework combining the International Classification of Diseases, 10th Revision (ICD-10) and the Chinese Expert Consensus on Diagnosis and Treatment of Acute Gastroenteritis (2013 Edition). Specific diagnostic criteria included: ① Typical gastrointestinal symptoms (abdominal pain, diarrhea, nausea, vomiting, or bloating); ② Symptom duration ≥ 2 days per episode or recurrence ≥ 2 times per year; ③ Exclusion of other gastrointestinal diseases (e.g., peptic ulcer, gastric cancer, irritable bowel syndrome) based on symptom description and medical history. ICD-10 coding was completed by trained public health physicians, following the process: ① Collecting self-reported symptom details (frequency, duration, severity); ② Classifying and coding according to ICD-10 criteria (e.g., K29.901 for acute gastritis, K52.901 for non-infectious gastroenteritis); ③ Double verification of coding results, with inconsistent cases reviewed by a third expert. For participants who had not sought medical care, diagnosis was based on symptom descriptions aligned with the Chinese Expert Consensus. Although the diagnostic framework combines ICD-10 and Chinese expert consensus, the lack of objective indicators (e.g., stool routine, blood tests, or imaging examinations) may lead to misclassification bias. Self-reported medical history may also be subject to recall bias, especially for chronic symptoms. In alignment with the cross-sectional study design, quantifiable and reproducible clinical and laboratory parameters were prioritized to minimize information bias.

### Baseline data collection

Epidemiologic data were collected through face-to-face interviews utilizing a comprehensive questionnaire, following the acquisition of written informed consent. The questionnaire encompassed sociodemographic factors (including but not limited to gender, age, education level, annual income, body mass index [BMI] and blood pressure), lifestyle choices (including but not limited to smoking habits, dietary patterns, and eating speed), as well as eating behaviors. Educational attainment was classified into two categories: “illiterate,” referring to individuals with no formal education; and “educated”, which includes those who have completed elementary school, middle school, high school, college or university education. Irregular eating is defined as either skipping meals or consuming food at inconsistent times. Participants reported their self-assessed eating speed using three possible responses: slow, medium, and fast. These assessments were based on the subjects' subjective evaluation of their own eating pace in comparison to that of others. The classification for a "salt-containing diet" corresponds to low, medium, and high salt intake levels—defined respectively as less than 1 teaspoon of salt (< 5.75 g/day), between 1–2 teaspoons of salt (5.75–11.5 g/day), and more than 2 teaspoons of salt (> 11.5 g/day) [6]. For participants who did not engage in cooking or were uncertain about their typical salt consumption levels, the assessment relied on the overall perceived saltiness of their usual diet along with personal preferences for salty foods, thus this classification may be inherently subjective. Details of tobacco and alcohol use included the average number of cigarettes smoked per day (1 pack has 20 cigarettes), units of alcohol consumed per day (1 unit is 8 g or 10 ml of pure alcohol), duration of the smoking/drinking habit (in years), and age of initiation of smoking/drinking, and cumulative consumption of tobacco/alcohol (in pack-years/unit-years) was also calculated. Dietary intake was estimated by using a validated qualitative food frequency questionnaire (FFQ) covering specific food items commonly used in the region (Supplementary material: English version of questionnaire). In order to calculate accurate frequencies, the questionnaire had to take into account the seasonality of some foods, which would affect the frequency of consumption throughout the year. Therefore, participants were asked how often they consumed these foods on a weekly, monthly/annual basis. They were also asked about the duration of this time, in months, that they would eat this food (e.g., year-round food for 12 months and seasonal food for 2 months). Using these data, we can normalize to “times per week”, averaged over the course of a year. The frequency categories are as follows: “never”, “less than once a week”, “once a week or more but less than three times a week” and "three times a week or more ". The reference standard for food frequency analysis is "average weekly consumption frequency", calculated as follows: ① For year-round staple foods (consumed for ≥ 10 months a year), the "weekly frequency" is used directly; ② For seasonal foods (consumed for < 10 months a year), the average weekly frequency is converted using the formula: "(Frequency per month × Number of consumption months)/12 × 4"; ③ Frequency categories are unified as "Never", " < 1 time/week", "1–2 times/week", and " ≥ 3 times/week" to ensure consistent analysis standards. Weight and height were collected according to a standardized procedure using a conventional scale and a weight gauge. Blood pressure was measured using a sphygmomanometer. Body mass index (BMI; weight in kilograms divided by the square of height in meters) was calculated on the basis of anthropometric measurements of weight (kg) and height (m), and then categorized according to the threshold values recommended by the National Health and Family Planning Commission of the People's Republic of China for the Chinese population, whereby < 18.5 kg/m^2^ was considered underweight, and 18.5 kg/m^2^ to 23.9 kg/m^2^ as normal, 24.0 kg/m^2^ to 27.9 kg/m^2^ as overweight, and 28.0 kg/m^2^ as obese. The reliability and validity of the FFQ were assessed as follows: the standardized-item Cronbach’s α coefficient was 0.858. The Kaiser–Meyer–Olkin measure of sampling adequacy was 0.876, and Bartlett’s test of sphericity was statistically significant (*P* < 0.001). These results indicate good internal consistency and satisfactory construct validity of the questionnaire.

### Statistical analysis

Unconditional univariate and multivariate logistic regression analyses were conducted to compute crude and adjusted odds ratios (ORs) along with their corresponding 95% confidence intervals (CIs). For dietary variables, only those with statistically significant crude ORs in the unconditional univariate logistic regression analyses were further included in the unconditional multivariate logistic regression models. In the multivariate logistic regression models, potential confounders were adjusted [7]. These confounders included sex, age, body mass index (BMI), educational attainment, annual income, and the number of cigarettes smoked per day and/or units of alcohol consumed per day. These selected confounding factors were identified based on previous experience, existing knowledge of esophageal cancer, and univariate logistic regression analysis [8]. Furthermore, the models were adjusted for age, gender, and BMI, given their potential to significantly influence socioeconomic status, the duration of smoking or drinking, and the volume of alcohol consumed per day. Similarly, linear trend tests were performed by incorporating the median value of each category from detailed smoking and drinking data as continuous variables within the models. Additionally, sensitivity analyses were performed by restricting the dataset to cases diagnosed using methods of higher diagnostic reliability (e.g., participants with available medical diagnostic reports), in order to evaluate the robustness of the results and reduce potential outcome misclassification. All data underwent a rigorous double-entry and validation process, initially in EpiData version 3.1 and subsequently in Microsoft Excel. For statistical analysis, SPSS software (version 27.0) was employed, while GraphPad Prism 10.5 was utilized for creating graphical representations. All significance tests were two-tailed, and a *P*-value of less than 0.05 was deemed statistically significant.

## Results

### Characteristics and lifestyles of subjects

The distribution of the 11,518 study participants (2,849 gastroenteritis cases and 8,669 normal controls) according to selected socio-demographic characteristics, lifestyle and dietary habits is shown in Table 1. Females were more represented than males in both gastroenteritis cases (65.8%) and controls (60.1%). Multifactorial logistic regression analysis showed that females had a greater risk of developing gastroenteritis compared with males (OR 1.37; 95% CI, 1.22–1.53). This study did not find a statistically significant difference in the risk of developing gastroenteritis between age groups. Multifactorial logistic regression analysis showed that the risk of gastroenteritis was 1.24 (OR 1.24; 95% CI, 1.13–1.37) and 1.45 (OR 1.45; 95% CI, 1.27–1.65) times higher for overweight and obese people than for underweight, respectively. The results of multivariate regression analyses showed that economic income level was significantly and positively associated with the risk of gastroenteritis. After multivariate adjustment, compared with the low-income group, the risk ratio (OR) of gastroenteritis for those with an annual per capita income of ≥ 10,000 yuan was 1.20 (95% CI: 1.05–1.37); and those with an annual per capita income of ≥ 15,000 yuan had a further elevated risk of developing the disease, with an OR value of 1.28 (95% CI: 1.09–1.50), which were all statistically significant (*P* < 0.05). This dose–response relationship suggests that the risk of gastroenteritis increases with increasing economic income. As shown in Table 1, multifactorial logistic regression analyses revealed significant associations between several lifestyle factors and the risk of developing gastroenteritis. Specifically, the risk of gastroenteritis was significantly increased by 29% (OR = 1.29, 95% CI: 1.11–1.51) in those who ate faster compared to those who ate slower. In addition, those with irregular diets had a 28% higher risk of the disease than those with regular diets (OR = 1.28, 95% CI: 1.05–1.55). Of note, the risk of gastroenteritis was 1.22 times higher in hypertensive than in non-hypertensive people (OR = 1.22, 95% CI: 1.11–1.34), an association that was statistically significant (*P* < 0.05). In addition, education level was not significant after multifactorial adjustment. The level of salt-containing dietary intake showed a significant dose–response relationship with the risk of gastrointestinal inflammation, and multifactorial logistic regression analysis showed a significantly increased risk of gastrointestinal inflammation among those with a moderately salty current diet compared to the light diet group (OR = 2.46, 95% CI: 2.16–2.80), and a further increased risk among those with a high salt diet (OR = 2.47, 95% CI: 2.15–2.85). Notably, the effect of ten years of salt-containing dietary intake was even more significant: risk ratio ratios were higher in those with moderately salty diets (OR = 2.59, 95% CI: 2.27–2.96) and in the high-salt diet group (OR = 2.83, 95% CI: 2.45–3.26) than in those with clean diets (*P* < 0.001). This result suggests that chronic high-salt diets may have a cumulative damaging effect on the gastrointestinal mucosa. The degree of dietary pungency was significantly and positively associated with the risk of gastrointestinal inflammation. Multifactorial analysis showed that current moderate (OR = 1.23, 95% CI: 1.12–1.35) and high spiciness (OR = 1.22, 95% CI: 1.06–1.40) consumers had significantly higher risk than those who did not consume spicy food. The effect of spicy diet ten years earlier was more significant (moderate: OR = 1.24, 95% CI: 1.13–1.37; high spiciness: OR = 1.32, 95% CI: 1.16–1.51), suggesting that there may be a cumulative effect of long-term spicy diet (*P* < 0.05). The analysis of fatty meat consumption showed a significant dose-dependent increase in risk (*P* < 0.001). Compared with the non-consumption reference group, the moderate intake group had a hazard ratio of 1.42 (95% CI: 1.29–1.56, *P* < 0.001), while the highest intake group demonstrated a further elevated risk of 1.90 (95% CI: 1.58–2.29, *P* < 0.001). The corresponding sensitivity analysis results are presented in Supplementary Table S1. In the sensitivity analysis, most associations remained consistent with the main findings; however, increasing age and higher education were positively associated with an increased risk of gastroenteritis.

**Table 1:** Characteristics and distribution of study subjects and ORs (and 95% CIs) for gastroenteritis according to sociodemographic features, lifestyle, and eating habits

	**Cases** **(*n* = 2849)** **n(%)**	**Controls** **(*n* = 8669)** **n(%)**	**Adjusted OR**^**a**^ **(95%CI**)	***P***	**Adjusted OR**^**b**^** (95%CI)**	***P***
Gender						
Male	973 (34.2)	3458 (39.9)	1.00 (reference)	-	1.00 (reference)	-
Female	1876 (65.8)	5211 (60.1)	1.26 (1.16–1.38)	< 0.001	1.37 (1.22–1.53)	< 0.001
Age			*P*_trend_	0.528	*P*_trend_	0.317
< 51	854 (30.0)	2515 (29.0)	1.00 (reference)	-	1.00 (reference)	-
51-	1099 (38.6)	3270 (37.7)	1.00 (0.90–1.11)	0.959	0.98 (0.88–1.09)	0.686
61-	896 (31.4)	2884 (33.3)	0.95 (0.85–1.06)	0.356	0.92 (0.82–1.03)	0.155
BMI			*P*_trend_	< 0.001	*P*_trend_	< 0.001
Normal	1288 (45.2)	4525 (52.2)	1.00 (reference)	-	1.00 (reference)	-
Underweight	44 (1.5)	117 (1.3)	1.33 (0.93–1.87)	0.118	1.30 (0.91–1.85)	0.150
Overweight	1107 (38.9)	3062(35.3)	1.27 (1.16–1.40)	< 0.001	1.24 (1.13–1.37)	< 0.001
Obesity	410 (14.4)	965 (11.1)	1.47 (1.13–1.68)	< 0.001	1.45 (1.27–1.65)	< 0.001
Annual income per capita (RMB)			*P*_trend_	< 0.001	*P*_trend_	< 0.001
≤ 5,000	638 (22.4)	2058 (23.8)	1.00 (reference)	-	1.00 (reference)	-
5,001-	1333 (46.8)	4368 (50.4)	0.99 (0.88–1.10)	0.782	0.99 (0.89–1.11)	0.885
10,001-	554 (19.4)	1443 (16.6)	1.20 (1.05–1.37)	0.008	1.20 (1.05–1.37)	0.007
15,001-	324 (11.4)	800 (9.2)	1.26 (1.08–1.48)	0.004	1.28 (1.09–1.50)	0.002
Eating speed			*P*_trend_	< 0.001	*P*_trend_	< 0.001
Slow	378 (13.3)	1154 (13.3)	1.00 (reference)	-	1.00 (reference)	-
Moderate	1877 (65.9)	6169 (71.2)	0.92 (0.81–1.05)	0.220	0.93 (0.82–1.05)	0.247
Fast	594 (20.8)	1346 (15.5)	1.31 (1.12–1.53)	< 0.001	1.29 (1.11–1.51)	0.001
Regularity of diet						
Regular diet	2697 (94.7)	8309 (95.8)	1.00 (reference)	-	1.00 (reference)	-
Irregular diet	152 (5.3)	360 (4.2)	1.29 (1.06–1.57)	0.011	1.28 (1.05–1.55)	0.015
High blood pressure						
No	1778 (62.4)	5863 (67.6)	1.00 (reference)	-	1.00 (reference)	-
Yes	1071 (37.6)	2806 (32.4)	1.22 (1.12–1.34)	< 0.001	1.22(1.11–1.34)	< 0.001
Education						
Illiterate	1197 (42.0)	3363 (38.8)	1.00 (reference)	-	1.00 (reference)	-
Educated	1652 (58.0)	5306 (61.2)	0.94 (0.85–1.04)	0.247	0.94 (0.85–1.04)	0.244
Salty diet			*P*_trend_	< 0.001	*P*_trend_	< 0.001
Low-salt	353 (12.4)	2334 (26.9)	1.00 (reference)	^−^	1.00 (reference)	^−^
Moderate-salt	1667 (58.5)	4265 (49.2)	2.50 (2.20–2.84)	< 0.001	2.46 (2.16–2.80)	< 0.001
High-salt	829 (29.1)	2070 (23.9)	2.55 (2.22–2.94)	< 0.001	2.47 (2.15–2.85)	< 0.001
Salty diet ten years ago			*P*_trend_	< 0.001	*P*_trend_	< 0.001
Low-salt	333 (11.7)	2351 (27.1)	1.00 (reference)	-	1.00 (reference)	-
Moderate-salt	1614 (56.6)	4210 (48.6)	2.63 (2.31–3.00)	< 0.001	2.59 (2.27–2.96)	< 0.001
High-salt	902 (31.7)	2108 (24.3)	2.91 (2.53–3.36)	< 0.001	2.83 (2.45–3.26)	< 0.001
Spicy diet			*P*_trend_	< 0.001	*P*_trend_	< 0.001
Non-spicy	980 (34.4)	3480 (40.1)	1.00 (reference)	^−^	1.00 (reference)	^−^
Moderate- spicy	1447 (50.8)	4033 (46.5)	1.24 (1.13–1.37)	< 0.001	1.23 (1.12–1.35)	< 0.001
High-spicy	422 (14.8)	1156 (13.3)	1.26 (1.11–1.45)	< 0.001	1.22 (1.06–1.40)	0.005
Spicy diet ten years ago			*P*_trend_	< 0.001	*P*_trend_	< 0.001
Non-spicy	925 (50.0)	3358 (39.0)	1.00 (reference)	-	1.00 (reference)	-
Moderate-spicy	433 (23.4)	4040 (46.9)	1.26 (1.14–1.38)	< 0.001	1.24 (1.13–1.37)	< 0.001
High-spicy	491 (26.6)	1221 (14.1)	1.37 (1.20–1.55)	< 0.001	1.32 (1.16–1.51)	< 0.001
Preference for fatty meats			*P*_trend_	< 0.001	*P*_trend_	< 0.001
Non-preference	950 (33.3)	3712 (42.8)	1.00 (reference)	-	1.00 (reference)	-
Moderate preference	1695 (59.5)	4532 (52.3)	1.46 (1.33–1.60)	< 0.001	1.42 (1.29–1.56)	< 0.001
High preference	204 (7.2)	425 (4.9)	1.96 (1.63–2.36)	< 0.001	1.90 (1.58–2.29)	< 0.001

^a^Adjusted for Gender, Age, BMI

^b^Adjusted for Gender, Age, BMI, Annual income per capita (RMB), Education, Number of cigarettes per day, alcohol units consumed per day

### Smoking and drinking

Table 2 shows the results of the ORs and their 95% CIs of smoking-related variables to the risk of gastroenteritis in the study population. Smoking more than 30 cigarettes per day was significantly associated with a 65% increase in the risk of gastroenteritis after adjusting for potential confounders (*P* = 0.003). Among all participants, the risk of gastroenteritis was increased by 41% (*P* < 0.001), 27% (*P* = 0.031), and 42% (*P* < 0.001) for those who smoked a cumulative total of 20, 30, and 40 or more packs of cigarettes, respectively, compared with nonsmokers. In addition, exposure to secondhand smoke due to household smoking was positively associated with a 27% increased risk of gastroenteritis (*P* < 0.001). Duration of smoking showed a significant dose–response relationship with the risk of developing gastroenteritis, and multifactorial regression analysis showed that compared with non-smokers, those who had smoked for < 20 years had a reduced risk (OR = 0.84, 95% CI: 0.71–1.00), whereas those who had smoked for 20–35 years had a significant increase in risk of 38% (OR = 1.38, 95% CI: 1.20–1.58), and those who had smoked for 35–50 years had an increased risk of 36% (OR = 0.001). 50-year smokers had no significant association with the risk of gastroenteritis. Multifactorial regression analysis showed that initial smokers aged 20–24 years had a significantly higher risk of gastroenteritis compared to non-smokers (OR = 1.47, 95% CI: 1.19–1.81, *P* < 0.001).

**Table 2:** ORs (and 95% CIs) for smoking-related variables with gastroenteritis

	**Cases** **(*n* = 2849)** **n(%)**	**Controls** **(*n* = 8669)** **n(%)**	**Adjusted OR**^**a**^ **(95%CI)**	***P***	**Adjusted OR**^**b**^**(95%CI)**	***P***
Number of cigarettes per day						
Nonsmoker	2039 (71.6)	6248 (72.1)	1.00 (reference)	-	1.00 (reference)	-
< 11	280 (9.8)	1035 (11.9)	0.97 (0.83–1.12)	0.644	0.96 (0.83–1.11)	0.600
11-	420 (14.7)	1108 (12.8)	1.35 (1.19–1.54)	< 0.001	1.33 (1.16–1.52)	< 0.001
21-	55 (1.9)	156 (1.8)	1.27 (0.93–1.74)	0.138	1.25 (0.91–1.72)	0.173
31-	55 (1.9)	122 (1.4)	1.69 (1.21–2.35)	0.002	1.65 (1.19–2.31)	0.003
*P*_trend_			< 0.001		< 0.001	
Cumulative amount of smoking (pack-years)						
Nonsmoker	2039 (71.6)	6248 (72.1)	1.00 (reference)	-	1.00 (reference)	-
< 20	366 (12.8)	1271 (14.7)	1.02 (0.90–1.17)	0.747	1.02 (0.89–1.16)	0.818
20-	154 (5.4)	379 (4.4)	1.44 (1.18–1.76)	< 0.001	1.41 (1.15–1.73)	0.001
30-	128 (4.5)	348 (4.0)	1.30 (1.05–1.61)	0.018	1.27 (1.02–1.58)	0.031
40-	162 (5.7)	423 (4.8)	1.45 (1.19–1.77)	< 0.001	1.42 (1.15–1.74)	0.001
*P*_trend_			< 0.001		< 0.001	
Family smoking (exposure to secondhand smoking)						
No	1417 (49.7)	4971 (57.3)	1.00 (reference)	-	1.00 (reference)	-
Yes	1432 (50.3)	3698 (42.7)	1.29 (1.18–1.41)	< 0.001	1.27 (1.17–1.39)	< 0.001
Duration of smoking(years)						
Nonsmoker	2039 (71.6)	6248 (72.1)	1.00 (reference)	-	1.00 (reference)	-
< 20	197 (6.9)	845 (9.7)	0.84 (0.71–0.99)	0.043	0.84 (0.71–1.00)	0.043
20-	386 (13.5)	964 (11.1)	1.39 (1.22–1.60)	< 0.001	1.38 (1.20–1.58)	< 0.001
35-	207 (7.3)	547 (6.3)	1.39 (1.16–1.66)	< 0.001	1.36 (1.13–1.63)	0.001
50-	20 (0.7)	65 (0.7)	1.19 (0.71–1.98)	0.510	1.17 (0.70–1.96)	0.540
*P*_trend_			< 0.001		< 0.001	
Age at start of smoking(years)						
Nonsmoker	2039 (71.6)	6248 (72.1)	1.00 (reference)	-	1.00 (reference)	-
25-	98 (3.4)	268 (3.1)	1.33 (1.04–1.70)	0.022	1.27 (0.99–1.63)	0.058
20-	153 (5.4)	366 (4.2)	1.51 (1.23–1.85)	< 0.001	1.47 (1.19–1.81)	< 0.001
< 20	559 (19.6)	1787 (20.6)	1.11 (0.99–1.25)	0.071	1.10 (0.98–1.24)	0.104
*P*_trend_			< 0.001		0.001	

^a^Adjusted for Gender, Age, BMI

^b^Adjusted for Gender, Age, BMI, Annual income per capita (RMB), Education, alcohol units consumed per day

After multivariate adjustment, Table 3 shows that alcohol units consumed per day, age at starting drinking, and cumulative amount of drinking were not significantly associated with the risk of developing gastroenteritis (*P* > 0.05). However, for duration of drinking, those with over 20 years of alcohol consumption showed 1.23 times higher risk compared to non-drinkers (OR = 1.23, 95% CI: 1.05–1.46, *P* < 0.05). The corresponding sensitivity analyses are shown in Supplementary Tables S2 and S3, with overall trends remaining consistent with the main findings.

**Table 3:** ORs (and 95% CIs) for alcohol-related variables with gastroenteritis

	**Cases** **(*n* = 2849)** **n(%)**	**Controls** **(*n* = 8669)** **n(%)**	**Adjusted OR**^**a**^ **(95%CI)**	***P***	**Adjusted OR**^**b**^** (95%CI)**	***P***
Alcohol units consumed per day						
Nondrinker	2331 (81.8)	7117 (82.1)	1.00 (reference)	-	1.00 (reference)	-
< 4	112 (3.9)	291 (3.4)	1.28 (1.02–1.60)	0.035	1.22 (0.97–1.53)	0.093
4-	250 (8.8)	798 (9.2)	1.11 (0.94–1.31)	0.208	1.04 (0.88–1.22)	0.670
8-	156 (5.5)	463 (5.3)	1.22 (1.00–1.49)	0.052	1.10 (0.90–1.36)	0.343
*P*_trend_			0.047		0.335	
Duration of drinking (years)						
Nondrinker	2331 (81.8)	7117 (82.1)	1.00 (reference)	-	1.00 (reference)	-
< 20	153 (5.4)	525 (6.1)	1.01(0.83–1.22)	0.947	0.96 (0.79–1.16)	0.672
20-	269 (9.4)	714 (8.2)	1.38(1.14–1.57)	< 0.001	1.23 (1.05–1.46)	0.012
35-	96 (3.4)	313 (3.6)	1.13(0.88–1.44)	0.345	1.03 (0.80–1.32)	0.818
*P*_trend_			0.005		0.069	
Age at starting drinking (years)						
Nondrinker	2331 (81.8)	7117 (82.1)	1.00 (reference)	-	1.00 (reference)	-
25-	45 (1.6)	152 (1.7)	0.87 (0.76–1.00)	0.045	0.93 (0.66–1.31)	0.671
20-	95 (3.3)	241 (2.8)	0.89 (0.63–1.27)	0.523	1.28 (0.99–1.66)	0.056
< 20	378 (13.2)	1159 (13.4)	1.28 (0.94–1.60)	0.131	1.08 (0.94–1.24)	0.282
*P*_trend_			0.021		0.197	
Cumulative amount of drinking (unit-years)						
Nondrinker	2331(81.8)	7117 (82.1)	1.00 (reference)	-	1.00 (reference)	-
< 40	86 (3.0)	270 (3.1)	1.05 (0.82–1.35)	0.683	1.02 (0.79–1.31)	0.903
40-	70 (2.5)	253 (2.9)	0.97 (0.74–1.28)	0.841	0.92 (0.70–1.21)	0.534
80-	81 (2.8)	226 (2.6)	1.28 (0.98–1.66)	0.073	1.20 (0.92–1.56)	0.188
120-	281 (9.9)	803 (9.2)	1.27 (1.09–1.50)	0.003	1.17 (0.99–1.37)	0.070
*P*_trend_			0.025		0.261	

^a^Adjusted for Gender, Age, BMI

^b^Adjusted for Gender, Age, BMI, Annual income per capita (RMB), Education, Number of cigarettes per day

### Dietary intake

**Fig. 1:**
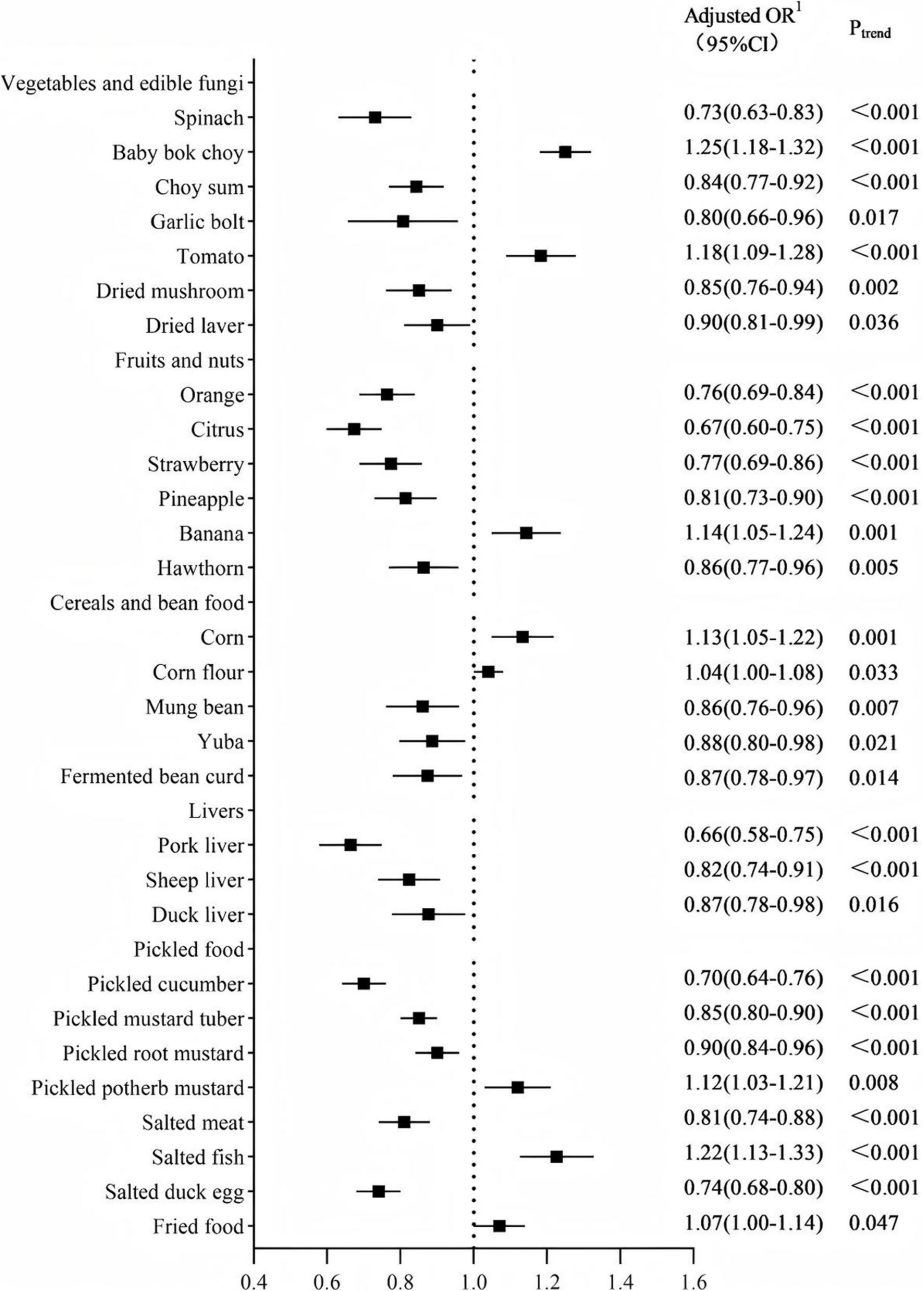
Association between dietary intake frequency and risk of gastroenteritis. Note: ^1^Adjusted for gender, age, BMI, annual income per capita (RMB), education, number of cigarettes per day, alcohol units consumed per day

The present study systematically assessed the association between eight broad categories of dietary factors (including: vegetables and edible mushrooms, fruits and nuts, cereals and legumes, liver, preserved foods, fried foods, and yeast products) and the risk of developing gastroenteritis. Variables showing statistical significance in the univariate logistic regression analyses for each food or food type (Supplementary Table S4) were subsequently included in the multivariate logistic regression model. After adjusting for potential confounders, the significant associations identified in this adjusted model are presented in Fig. 1. Foods significantly associated with reduced risk of gastroenteritis included certain types of vegetables, edible mushrooms, fruits, legumes, and liver foods. In addition, foods significantly associated with an increased risk of gastroenteritis included baby bok choy, tomatoes, bananas, maize, cornmeal, pickled potherbs, salted fish, and fried foods. The corresponding sensitivity analysis results are presented in Supplementary Figure S1. Overall, the results are consistent, although some individual data points differ, such as for pickled mustard tuber.

## Discussion

### Sociodemographics

This study found that the incidence of gastroenteritis was significantly higher in females than in males, which was consistent with the findings of many epidemiological studies at home and abroad [9–11], and the potential mechanisms of its influence are as follows: from the physiological point of view, the cyclic changes of estrogen and progesterone unique to females can change gastrointestinal dynamics, affect the function of the mucosal barrier, and regulate the composition of the intestinal flora, and the dramatic fluctuation of hormone levels in the perimenopausal period may further increase the susceptibility to gastroenteritis [12]. In terms of psychoneurological mechanisms, the higher prevalence of anxiety, depression and other psychological disorders in the female population may affect intestinal function through the brain-gut axis, and chronic stress may lead to increased intestinal permeability and immune dysfunction, which may increase the risk of developing gastroenteritis [10, 13, 14].In addition, differences in health behaviors are also an important factor, as women's higher utilization of healthcare services and reporting of symptoms may lead to diagnostic bias [15], and their dominant role in home food preparation may also be an important factor. The incidence rates of gastroenteritis in this study did not show statistically significant differences between different age groups, which is different from some previous studies, and the possible reasons include: the incidence of gastroenteritis is affected by a combination of factors such as environmental exposure, lifestyle and co-morbidities, etc. [16, 17], and although there are physiological changes in the elderly group such as declining immune function and weakening of the mucosal barrier [18, 19], the age-related risk may be offset in the elderly group of the present study by the health management strategies such as a regular diet and the rational use of medication [20]. At the same time, it is difficult to capture the long-term trend of gastroenteritis as an acute or recurrent disease in a cross-sectional study design, and its short-term fluctuating characteristics may mask the cumulative effect of age.

Interestingly, the positive association between higher income levels and gastroenteritis risk observed in this study contrasts with the traditional concept of a “disease-poverty correlation.” This counterintuitive finding may reflect complex lifestyle and behavioral transitions accompanying socioeconomic improvement in rural areas. This association may partly be explained by dietary transitions among higher-income groups, including increased consumption of high-fat, high-protein, and processed foods, which has been linked to alterations in gut microbiota composition and a pro-inflammatory milieu characterized by elevated levels of inflammatory mediators such as TNF-α and IL-6 [21]. Moreover, a higher frequency of eating out may reflect less regulated dietary practices and greater reliance on restaurant-prepared or processed foods, which have been associated with unfavorable gastrointestinal health outcomes [22]. In addition, frequent eating out may increase exposure to foodborne pathogens, and when combined with greater health awareness and healthcare-seeking behavior, may contribute to higher disease detection rates [23].

### Lifestyle

The present study demonstrated that irregular diet and rapid eating rate significantly increased the risk of gastroenteritis. Using a retrospective dietary assessment method, this study investigated, for the first time, the long-term effects of current and 10-year prior sodium and capsaicin exposure on gastroenteritis. We found that cumulative exposure to high-sodium and spicy diets significantly increased the risk of gastroenteritis, consistent with previous findings [24–27]. Notably, long-term high-salt intake emerged as a particularly important risk factor. Mechanistically, excessive dietary salt may damage gastric and intestinal mucosa, disrupt tight junction integrity, and promote mucosal inflammation [28, 29]. Moreover, chronic high-salt consumption may alter gut microbiota composition, favoring pro-inflammatory microbial profiles and thereby enhancing susceptibility to gastrointestinal injury [29, 30].

Although a causal association between smoking and gastroenteritis has been reported in previous studies [31, 32], the present study showed that most smoking-related variables were significantly associated with an increased risk of gastroenteritis, which is consistent with studies suggesting that cumulative tobacco exposure and current smoking behaviors may play a more prominent role in gastrointestinal injury. In addition to active smoking, the present study also observed a positive association between exposure to second-hand smoke and the risk of gastroenteritis. Passive exposure to tobacco smoke has been shown to induce systemic and gastroesophageal inflammation, increase oxidative stress, and impair mucosal defense mechanisms [33]. Evidence suggests that cigarette smoking or second-hand smoke can alter gut motility, disrupt intestinal barrier integrity, and modulate the gut microbiota, thereby increasing susceptibility to gastrointestinal disorders [34, 35]. In rural households, exposure to second-hand smoke often occurs in enclosed domestic environments and may be sustained over long periods, particularly among children and women [36, 37]. This finding highlights second-hand smoke as an important but underrecognized environmental risk factor for gastroenteritis and underscores the need for smoke-free household interventions in rural communities. No statistically significant association was found between alcohol consumption and gastroenteritis, which needs to be validated with large samples and accurate exposure assessment.

Psychological stress is an important influencing factor for gastrointestinal diseases [11, 12], which may affect intestinal permeability and gut microbiota balance through the gut-brain axis. However, this study did not collect stress-related data (e.g., life stress events, anxiety and depression status), so stress could not be incorporated into the adjustment model, potentially leading to residual confounding. We recommend that future studies include stress as a factor in the analyses.

### Dietary intake

Intake of fresh fruits and vegetables has long been thought to be negatively associated with the risk of gastroenteritis [38, 39]. Our analyses included the most commonly consumed fresh fruits and vegetables in Huai'an. The final adjusted results showed that high intake of edible mushrooms and non-starchy vegetables, such as spinach, greens, and garlic scapes, were statistically significantly associated with a reduced risk of gastroenteritis. Whereas most fruits intake showed a protective effect. Antioxidant micronutrients such as vitamins and minerals are thought to potentially confer anticancer activity to fruits and vegetables [40], but few published papers have examined the relationship between fruit and vegetable intake and risk of gastroenteritis. Figure 1 also shows that increased consumption of mung beans, adzuki beans, curd and animal liver was negatively associated with the risk of gastroenteritis. It has long been believed that the potential association between consumption of these foods and reduced gastroenteritis risk may be related to their richness in antioxidant micronutrients and some bioactive compounds such as fiber, vitamins A, C, E, B vitamins, selenium, polyphenols, phytosterols, flavonoids and isoflavones [41–43]. The protective effect observed with the intake of animal liver may be due to its richness in minerals, vitamin A and B vitamins, such as folic acid; also, folic acid, vitamin A and its precursor b-carotene have been shown to play a preventive role in a number of diseases, including gastroenteritis [44]. A systematic comparative study of the chemicals and antioxidant capacity of 13 types of edible legumes showed that pinto beans had the strongest oxygen radical uptake capacity, followed by mung beans, and pinto and mung beans ranked second and sixth in peroxyl radical scavenging capacity [45]. Pinto and mung beans showed excellent antioxidant activity in vitro, with both containing much higher levels of total phenols and proanthocyanidins than most other legumes [45]. The fermentation process provided enhanced antioxidant and antiproliferative activities in soy foods because of the changes in isoflavone composition during curd fermentation, particularly the conversion of isoflavones from glycoside couplings to glycoside forms with higher bioavailability [46].The results of dietary factors in this study showed that the risk of gastroenteritis was associated with increased intake of chard, tomatoes, bananas, maize, corn flour, fried foods, and pickled/salted, such as pickled potherbs. Intake of fried foods is a well-known risk factor for gastroenteritis [47, 48]. It was suggested that consumption of pickled foods is very common and may be one of the risk factors for gastroenteritis in high-risk areas of China [49]. Contamination of n-nitroso compounds such as n-nitrosamines in pickled foods has been shown to be an important mechanism, as well as other compounds such as Roussin red methyl ester and mycotoxins released by microorganisms, which have also been implicated in the carcinogenicity of these foods [50, 51]. In this region, harvested maize is essentially stored by solar dehydration. However, the known carcinogens aflatoxin and fumonisin are frequently produced in stored maize and flour, thus posing a risk to human health. Previous studies by our group confirmed the contamination of two mycotoxins in maize from Huai'an and suggested that they play a contributory role in esophageal carcinogenesis [52, 53]. Therefore, consumption of this contaminated maize and maize flour as a staple food may be a risk factor in Huai'an. Stir-frying, deep-frying and pan-frying are common cooking methods in China that involve heating oil to high temperatures. These cooking methods produce carcinogens such as genotoxic heterocyclic amines, benzo[a]pyrene, formaldehyde, and benzene in the emissions and edible portions of food, especially when frying protein-rich foods [54–56]. Pickled foods, which are very popular in Huai'an, are associated with an increased risk of gastroenteritis. Similar findings were observed in a previous study on the adverse effects of pickled vegetables on gastroenteritis [57]. Contamination of n -nitroso compounds such as n -nitrosamines in pickled foods has been shown to be an important mechanism, as well as other compounds such as Roussin red methyl ester and mycotoxins released by microorganisms, which have also been implicated in the hazards of these foods [58].

This study has several limitations. First, limitation of the study design: As a cross-sectional study, it can only reveal associations rather than establish causal relationships. It fails to capture the long-term dynamic changes of gastroenteritis, which may mask the cumulative effects of factors such as age and diet. For future research, prospective cohort studies are recommended to track the time-series relationship between exposures and outcomes. Second, this study’s sample was derived from the “Early Diagnosis and Early Treatment Project of Esophageal Cancer” in rural Huai’an, Jiangsu Province, with participants being permanent residents aged 35–75 years. The project covers a limited area, only representing rural regions of Huai’an and thus cannot be generalized to other rural areas in China (e.g., rural areas in northern or western China); Participants were voluntary screening attendees, who may pay more attention to health than the general rural population, resulting in “healthy volunteer bias”; Migrant populations, bedridden individuals, and other special groups were not included, limiting the representativeness of the sample. Future studies should expand the sample coverage and adopt a multicenter sampling design to enhance the external validity of the results. Third, limitation of diagnostic methods: As this study is a secondary analysis, objective diagnostic indicators for gastroenteritis were not consistently available during follow-up of the whole study, and some gastroenteritis cases relied on self-reported status, which may have introduced diagnostic misclassification. Sensitivity analyses restricted to cases with more accurate diagnostic methods showed that the results did not change significantly. However, some results may differ in analyses limited to cases diagnosed using high-reliability methods, as the number of such cases (n = 300) is smaller than that in the primary analysis. Future studies should incorporate simple, objective assessments (e.g., routine stool tests or H. pylori testing) to improve the accuracy and consistency of case definitions. Fourth, limitation of confounding factors: Failure to include important confounding factors such as Helicobacter pylori (Hp) infection, psychological stress, and family hygiene conditions may result in resultant bias. Future research should comprehensively collect relevant data to optimize the adjustment model. Fifth, limitation of dietary assessment: Dietary classification relies on subjective judgment with insufficient quantitative precision, and the FFQ cannot capture short-term dietary changes. Future studies may combine the 24-h dietary recall method and food diaries to enhance the accuracy of dietary exposure assessment. Sixth, limitation of variable selection: The selection of adjustment variables is based on existing data and literature, and there may be unidentified confounding factors. Future studies could adopt methods such as propensity score matching and instrumental variables to further control for residual confounding.

## Conclusions

Various factors, including dietary habits, lifestyle, and dietary intake, were identified as relevant to the study population. However, certain factors, such as age and alcohol consumption, require further investigation to elucidate their precise impact on gastroenteritis incidence. Our findings underscore the urgent need for targeted policies and interventions to address modifiable risk factors among rural populations in China. These results contribute to the growing body of literature on gastrointestinal epidemiology and its associated risk factors, particularly in low socioeconomic settings, while highlighting the importance of tailored public health measures to mitigate gastrointestinal diseases in these communities.

## Supplementary Information

Supplementary Material 1.

Supplementary Material 2.

## Data Availability

All data generated or analyzed during this study are included. The technical appendix and statistical procedure are available from the corresponding author upon reasonable request.
