# Supplementary material for: Gastroenteritis in middle-aged and elderly adults in rural China: associations with sociodemographic, lifestyle, and dietary factors
Source: BMC Gastroenterol. 2026 May 18;26:427. doi: 10.1186/s12876-026-04916-0 (PMC13348955; doi:10.1186/s12876-026-04916-0)
Supplement: Supplementary file 1 — Supplementary Material 1. [file 12876_2026_4916_MOESM1_ESM.pdf]

Name______________ Study ID#|__|__|__|__|__|__|__|__|__|__|__|__|

Endoscopy Examination and Treatment Informed Consent for the Early Diagnosis and Early Treatment Project of Esophageal Cancer

Esophageal cancer is a common disease in this area, and many local residents die from esophageal cancer due to late treatment. The purpose of this examination is to detect the cancer early and treat it promptly, achieving long-term survival outcomes. Additionally, early detection, diagnosis, and treatment through screening will help summarize experiences for nationwide promotion, quickly reduce esophageal cancer mortality, and effectively control the harm caused by esophageal cancer in high-risk areas, benefiting the public.

Examination and Treatment Process

We invite local residents aged 40-69 to participate in endoscopic examinations. In addition to visual observation during the endoscopy, tissue samples will be taken from the affected areas for accurate diagnosis. If esophageal cancer or precancerous lesions (such as severe atypical hyperplasia) are detected, different treatments will be arranged based on the individual's condition. Endoscopic mucosal resection may be performed for individuals with severe atypical hyperplasia or early-stage cancer. Surgery or radiotherapy may be considered for individuals with mid-to-late stage cancer.

Risks of Participating in the Examination and Treatment

Endoscopic examination and treatment are generally safe. However, in rare cases, complications such as bleeding, esophageal perforation, and drug reactions (e.g., iodine allergy) may occur. Doctors will take preventive measures, and even if complications arise, they will be promptly handled. However, serious situations may occur, which could even pose a life-threatening risk. If you wish to learn more about these risks, please contact the medical staff in the examination team.

Benefits of Participating in the Examination

By participating in the examination, you will gain a comprehensive understanding of the condition of your esophagus, stomach, and duodenum. If tumors are detected, they can be treated promptly.

Confidentiality

Your examination results will be strictly protected according to relevant laws and will not be disclosed. Biopsy specimens collected during the project will be stored and may be used in future research. These biopsy specimens will be labeled with coded tags, and your personal information will be kept confidential.

Voluntary Participation

Participation in the examination is entirely voluntary, and the decision to participate is yours to make. You can refuse to participate and have the right to withdraw from the examination at any time. If you have any questions, please contact the examination team. Phone: ____________________; Address: ____________________.

Self-Declaration

I have read this informed consent form and understand all the information. Some questions have been discussed with the examination team, and I am satisfied with the answers. I agree to participate in the endoscopic examination and treatment.

Participant's Signature: _________________ Date: _________________

Witness Declaration

I have read and explained this informed consent form to the participant. The participant has understood and agreed to participate in this project.

Witness Signature: _________________ Date: _________________

Early Diagnosis and Early Treatment Project of Esophageal Cancer Baseline Survey

Name:____________________ Study ID# |__|__|__|__|__|__|__|__|__|__|__|__|

Sex:(Male--1,Female--2) |__| Date of Birth: ____________________

ID Number: |__|__|__|__|__|__|__|__|__|__|__|__|__|__|__|__|__|__|

Survey Date: ____________________ Surveyor's Name:___________

1.General Information：

1.1 Marital Status： |___|
(0 = Unmarried, 1 = Married, 2 = Divorced, 3 = Widowed)

1.2 Highest Education Level： |___|
(0 = Never attended school, 1 = Primary school, 2 = Middle school, 3 = College or above)

1.3 Personality Type： |___|
(1 = Outgoing, 2 = Fairly outgoing, 3 = Introverted)

1.4 Social Relationships： |___|
(1 = Good, 2 = Average, 3 = Poor, 4 = Very poor)

1.5 Do you have regular meals three times a day： |___|
(0 = No, 1 = Yes)

1.6 Compared to the general population, your usual eating speed is： |___|
(1 = Fast, 2 = Moderate, 3 = Slow)

1.7 Your usual saltiness in food is： |___|
(1 = Very salty, 2 = Quite salty, 3 = Not too salty, 4 = Not salty)

1.8 Ten years ago, your usual saltiness in food was： |___|
(1 = Very salty, 2 = Quite salty, 3 = Not too salty, 4 = Not salty)

1.9 Your usual spiciness in food is： |___|
(1 = Very spicy, 2 = Quite spicy, 3 = Not too spicy, 4 = Not spicy)

1.10 Ten years ago, your usual spiciness in food was： |___|
(1 = Very spicy, 2 = Quite spicy, 3 = Not too spicy, 4 = Not spicy)

1.11 Your usual consumption of meat dishes is： |___|
(0 = Do not eat meat, 1 = All fatty meat, 2 = Some fatty meat, 3 = No fatty meat)

1.12 Do the grains stored in your home have mold： |___|
(0 = No, 1 = Yes)

1.13 Ten years ago, did the grains stored in your home have mold： |___|
(0 = No, 1 = Yes)

1.14 How many people are in your family, and what is your average annual income?
|__|__|people， |__|__|__|__|__|__|Yuan/Year

2. Water Consumption:

2.1 Your main source of drinking water is： |___|
(1 = Shallow well water, 2 = Lake water, river water, 3 = Deep well water, 4 = Tap water (treated))

3. Smoking: (0 = No, 1 = Yes)

Type	Yes/No	Daily/Monthly Quantity	Total Years of Smoking
3.1 Cigarettes	|__|	|__|__|Cigarettes /day	|__|__|Years
3.2 Loose tobacco	|__|	|__|__|Taels/month	|__|__| Years

3.3 Do any of your family members (parents, spouse, relatives, housemates) smoke： |___|
(0=否, 1=是)

4. Drinking: (0 = No, 1 = Yes)

Type	Yes/No	Daily Quantity	Total Years of Drinking
4.1 Beer	|__|	|__|__|__|__|__|mL/day	|__|__|Years
4.2 Liquor	|__|	|__|__|Taels/day	|__|__|Years
4.3 Fruit wine	|__|	|__|__|Taels/day	|__|__|Years
4.4 Other alcohol	|__|	|__|__|Taels/day	|__|__|Years

5. Tea Consumption: (0 = No, 1 = Yes)

Type	Yes/No	Daily Quantity	Total Years of

			Drinking
5.1 Flower tea	|__|	|__|Taels/month	|__|__|Years
5.2 Green tea	|__|	|__|Taels/month	|__|__|Years
5.3 Black tea	|__|	|__|Taels/month	|__|__|Years

6. Dietary Habits: (In the past year, have you consumed the following foods? Please fill in the corresponding columns with numbers)

Food Name	Times/week	Times/month	Times/year	Duration (Months)
6.1 Fresh vegetables	|__|__|	|__|	|__|__|	|__|__|
Coriander	|__|__|	|__|	|__|__|	|__|__|
Chrysanthemum coronarium	|__|__|	|__|	|__|__|	|__|__|
Amaranthustricolor	|__|__|	|__|	|__|__|	|__|__|
Spinach	|__|__|	|__|	|__|__|	|__|__|
Baby bok choy	|__|__|	|__|	|__|__|	|__|__|
Garlic chives	|__|__|	|__|	|__|__|	|__|__|
Rapeseed	|__|__|	|__|	|__|__|	|__|__|
Garlic bolt	|__|__|	|__|	|__|__|	|__|__|
Lettuce	|__|__|	|__|	|__|__|	|__|__|
Tomato	|__|__|	|__|	|__|__|	|__|__|
6.2 Fresh fruits	|__|__|	|__|	|__|__|	|__|__|
Orange	|__|__|	|__|	|__|__|	|__|__|
Citrus	|__|__|	|__|	|__|__|	|__|__|
Strawberry	|__|__|	|__|	|__|__|	|__|__|
Pineapple	|__|__|	|__|	|__|__|	|__|__|
Banana	|__|__|	|__|	|__|__|	|__|__|
Hawthorn	|__|__|	|__|	|__|__|	|__|__|
6.3 Livers	|__|__|	|__|	|__|__|	|__|__|
Pork liver	|__|__|	|__|	|__|__|	|__|__|
Sheep liver	|__|__|	|__|	|__|__|	|__|__|
Chicken liver	|__|__|	|__|	|__|__|	|__|__|
Duck liver	|__|__|	|__|	|__|__|	|__|__|
6.4 Bean food and legumes	|__|__|	|__|	|__|__|	|__|__|
Soy bean	|__|__|	|__|	|__|__|	|__|__|
mung bean	|__|__|	|__|	|__|__|	|__|__|
Adzuki bean	|__|__|	|__|	|__|__|	|__|__|
Yuba	|__|__|	|__|	|__|__|	|__|__|
6.5 Garlic, onions	|__|__|	|__|	|__|__|	|__|__|
6.6 Staple food	|__|__|	|__|	|__|__|	|__|__|
Corn	|__|__|	|__|	|__|__|	|__|__|
Corn flour	|__|__|	|__|	|__|__|	|__|__|
6.7 Nuts and dried vegetables	|__|__|	|__|	|__|__|	|__|__|
Peanut	|__|__|	|__|	|__|__|	|__|__|
Walnut	|__|__|	|__|	|__|__|	|__|__|
Dried mushroom	|__|__|	|__|	|__|__|	|__|__|
Dried laver	|__|__|	|__|	|__|__|	|__|__|
6.8 Pickled food	|__|__|	|__|	|__|__|	|__|__|
Pickled radish	|__|__|	|__|	|__|__|	|__|__|
Pickled cucumber	|__|__|	|__|	|__|__|	|__|__|
Pickled mustard tuber	|__|__|	|__|	|__|__|	|__|__|
Pickled root mustard	|__|__|	|__|	|__|__|	|__|__|
Pickled potherb mustard	|__|__|	|__|	|__|__|	|__|__|
Salted meat	|__|__|	|__|	|__|__|	|__|__|
Salted fish	|__|__|	|__|	|__|__|	|__|__|
Salted duck egg	|__|__|	|__|	|__|__|	|__|__|
Fermented bean curd	|__|__|	|__|	|__|__|	|__|__|
6.9 Fried food	|__|__|	|__|	|__|__|	|__|__|
6.10 Hot diet	|__|__|	|__|	|__|__|	|__|__|
6.11 Yeast	|__|__|	|__|	|__|__|	|__|__|

7. Digestive System Disease History: (0 = No, 1 = Yes)

Disease Name	Yes/No	Age at Diagnosis
7.1 Gastroenteritis	|__|	|__|__| Years
7.2 Gastric or duodenal ulcers	|__|	|__|__| Years
7.3 Esophagitis	|__|	|__|__| Years
7.4 Hepatitis	|__|	|__|__| Years
7.5 Other (Please specify)		|__|__| Years

8. Family History: (Has anyone in your family had cancer, 0 = No, 1 = Yes) |__|

Relative	Type of Cancer	Number of People

9. Physical Examination Results:

Height (cm)	Weight (kg)	Pulse (beats/min)	Blood Pressure (mmHg)
|__|__|__|	|__|__|__|	|__|__|__|	|__|__|__|/|__|__|__|
