# Supplementary material for: Gastroenteritis in middle-aged and elderly adults in rural China: associations with sociodemographic, lifestyle, and dietary factors
Source: BMC Gastroenterol. 2026 May 18;26:427. doi: 10.1186/s12876-026-04916-0 (PMC13348955; doi:10.1186/s12876-026-04916-0)
Supplement: Supplementary file 2 — Supplementary Material 2. [file 12876_2026_4916_MOESM2_ESM.docx]

| **Table S1.** Sensitivity analysis of characteristics and distribution of study subjects and ORs (95% CIs) for gastroenteritis according to sociodemographic features, lifestyle, and eating habits, restricted to cases diagnosed using high-reliability methods. | | | | | | | |
| --- | --- | --- | --- | --- | --- | --- | --- |
|  | **Cases  (n=300)  n(%)** | **Controls （n=8088）  n(%)** | **Adjusted OR^a^**  **（95%CI**） | ***P*** | **Adjusted OR^b^ (95%CI)** | ***P*** |  |
| Gender |  |  |  |  |  |  |  |
| Male | 122 (40.7) | 3196 (39.5) | 1.00 (reference) | - | 1.00 (reference) | - |  |
| Female | 178 (59.3) | 4892 (60.5) | 1.09 (0.94-1.26) | 0.673 | 1.09 (0.81-1.46) | 0.578 |  |
| Age |  |  | *P*_trend_ | 0.062 | *P*_trend_ | 0.005 |  |
| <51 | 72 (24.0) | 2377 (29.4) | 1.00 (reference) | - | 1.00 (reference) | - |  |
| 51- | 130 (43.3) | 3040 (37.6) | 1.42 (1.06-1.90) | 0.019 | 1.64 (1.21-2.22) | 0.001 |  |
| 61- | 98 (32.7) | 2671 (33.0) | 1.22 (0.89-1.66) | 0.219 | 1.51 (1.08-2.10) | 0.015 |  |
| BMI |  |  | *P*_trend_ | <0.001 | *P*_trend_ | 0.003 |  |
| Normal | 128 (42.7) | 4299 (53.15) | 1.00 (reference) | - | 1.00 (reference) | - |  |
| Underweight | 2 (0.01) | 108 (1.34) | 0.61 (0.15-2.50) | 0.492 | 0.64 (0.16-2.63) | 0.536 |  |
| Overweight | 121 (40.3) | 2806 (34.69) | 1.45 (1.13-1.87) | 0.004 | 1.39 (1.07-1.79) | 0.013 |  |
| Obesity | 49 (16.3) | 875 (10.82) | 1.89 (1.35-2.65) | <0.001 | 1.79 (1.27-2.52) | <0.001 |  |
| Annual income per capita (RMB) |  |  | *P*_trend_ | <0.001 | *P*_trend_ | <0.001 |  |
| ≤5,000 | 45 (15.00) | 1955 (24.17) | 1.00 (reference) | - | 1.00 (reference) | - |  |
| 5,001- | 136 (45.33) | 4104 (50.74) | 1.49 (1.06-2.10) | 0.023 | 1.45 (1.03-2.04) | 0.034 |  |
| 10,001- | 68 (22.67) | 1303 (16.11) | 2.23 (1.52-3.29) | <0.001 | 2.18 (1.48-3.21) | <0.001 |  |
| 15,001- | 51 (17.00) | 726 (8.98) | 3.02 (1.99-4.57) | <0.001 | 2.93 (1.93-4.44) | <0.001 |  |
| Eating speed |  |  | *P*_trend_ | 0.016 | *P*_trend_ | 0.031 |  |
| Slow | 36 (12.0) | 1095 (13.54) | 1.00 (reference) | - | 1.00 (reference) | - |  |
| Moderate | 197 (65.67) | 5753 (71.13) | 1.01 (0.71-1.46) | 0.942 | 0.99 (0.69-1.42) | 0.945 |  |
| Fast | 67 (22.33) | 1240 (15.33) | 1.53 (1.01-2.33) | 0.047 | 1.45 (0.95-2.21) | 0.084 |  |
| Regularity of diet |  |  |  |  |  |  |  |
| Regular diet | 288 (96.0) | 7758 (95.9) | 1.00 (reference) | - | 1.00 (reference) | - |  |
| Irregular diet | 12 (4.0) | 330 (4.1) | 0.97 (0.54-1.75) | 0.914 | 0.99 (0.55-1.79) | 0.981 |  |
| High blood pressure |  |  |  |  |  |  |  |
| No | 202 (67.3) | 5517 (68.2) | 1.00 (reference) | - | 1.00 (reference) | - |  |
| Yes | 98 (32.7) | 2571 (31.8) | 0.90 (0.69-1.16) | 0.404 | 0.93(0.72-1.20) | 0.566 |  |
| Education |  |  |  |  |  |  |  |
| Illiterate | 102 (34.0) | 3167 (39.2) | 1.00 (reference) | - | 1.00 (reference) | - |  |
| Educated | 198 (66.0) | 4921 (60.8) | 1.43 (1.08-1.90) | 0.012 | 1.37 (1.03-1.81) | 0.030 |  |
| Salty diet |  |  | *P*_trend_ | <0.001 | *P*_trend_ | <0.001 |  |
| Low-salt | 27 (9.0) | 2274 (28.1) | 1.00 (reference) | ^-^ | 1.00 (reference) | ^-^ |  |
| Moderate-salt | 173 (57.7) | 3916 (48.4) | 3.45 (2.28-5.23) | <0.001 | 3.44 (2.26-5.22) | <0.001 |  |
| High-salt | 100 (33.3) | 1898 (23.5) | 4.10 (2.64-6.34) | <0.001 | 4.10 (2.63-6.37) | <0.001 |  |
| Salty diet ten years ago |  |  | P_trend_ | <0.001 | *P*_trend_ | <0.001 |  |
| Low-salt | 25 (8.3) | 2289 (28.3) | 1.00 (reference) | - | 1.00 (reference) | - |  |
| Moderate-salt | 166 (55.4) | 3883 (48.0) | 3.66 (2.38-5.63) | <0.001 | 3.67 (2.38-5.66) | <0.001 |  |
| High-salt | 109 (36.3) | 1916 (23.7) | 4.83 (3.09-7.56) | <0.001 | 4.84 (3.08-7.60) | <0.001 |  |
| Spicy diet |  |  | *P*_trend_ | 0.028 | *P*_trend_ | 0.021 |  |
| Non-spicy | 95 (31.7) | 3321 (41.1) | 1.00 (reference) | ^-^ | 1.00 (reference) | ^-^ |  |
| Moderate- spicy | 162 (54.0) | 3710 (45.9) | 1.43 (1.10-1.86) | 0.008 | 1.45 (1.12-1.89) | 0.006 |  |
| High-spicy | 43 (14.3) | 1057 (13.0) | 1.31 (0.90-1.91) | 0.152 | 1.35 (0.92-1.97) | 0.127 |  |
| Spicy diet ten years ago |  |  | *P*_trend_ | 0.023 | *P*_trend_ | 0.018 |  |
| Non-spicy | 90 (30.0) | 3206 (36.4) | 1.00 (reference) | - | 1.00 (reference) | - |  |
| Moderate-spicy | 159 (53.0) | 3720 (42.2) | 1.43 (1.10-1.87) | 0.009 | 1.45 (1.11-1.90) | 0.007 |  |
| High-spicy | 51 (17.0) | 1162 (13.2) | 1.44 (1.01-2.06) | 0.046 | 1.48 (1.03-2.13) | 0.035 |  |
| Preference for fatty meats |  |  | *P*_trend_ | <0.001 | *P*_trend_ | <0.001 |  |
| Non-preference | 85 (28.3) | 3522 (43.5) | 1.00 (reference) | - | 1.00 (reference) | - |  |
| Moderate preference | 197 (65.7) | 4164 (51.5) | 1.82 (1.40-2.37) | <0.001 | 1.78 (1.36-2.32) | <0.001 |  |
| High preference | 18 (6.0) | 402 (5.0) | 1.71 (1.01-2.90) | 0.045 | 1.78 (1.05-3.02) | 0.033 |  |
| ^a^ Adjusted for Gender, Age, BMI. | | | | | | | |
| ^b^ Adjusted for Gender, Age, BMI, Annual income per capita (RMB), Education, Number of cigarettes per day, alcohol units consumed per day. | | | | | | | |

| **Table S2.** Sensitivity analysis of ORs (95% CIs) for smoking-related variables with gastroenteritis, restricted to cases diagnosed using high-reliability methods. | | | | | | |  |
| --- | --- | --- | --- | --- | --- | --- | --- |
|  | **Cases  (n=300)  n(%)** | **Controls （n=8088）  n(%)** | **Adjusted OR^a^ （95%CI）** | ***P*** | **Adjusted OR^b^（95%CI）** | ***P*** | |
| Number of cigarettes per day |  |  |  |  |  |  | |
| Nonsmoker | 219 (73.0) | 5830 (72.1) | 1.00 (reference) | - | 1.00 (reference) | - | |
| <11 | 18 (6.0) | 978 (12.1) | 0.50 (0.30-0.82) | 0.006 | 0.51 (0.31-0.84) | 0.008 | |
| 11- | 53 (17.7) | 1022 (12.6) | 1.31 (0.94-1.82) | 0.108 | 1.41 (1.00-1.98) | 0.048 | |
| 21- | 4 (1.3) | 144 (1.8) | 0.70 (0.25-1.92) | 0.483 | 0.77 (0.28-2.14) | 0.618 | |
| 31- | 6 (2.0) | 114 (1.4) | 1.29 (0.55-3.01) | 0.559 | 1.48 (0.63-3.50) | 0.370 | |
| *P*_trend_ |  |  | 0.011 |  | 0.006 |  | |
| Cumulative amount of smoking (pack-years) | |  |  |  |  |  | |
| Nonsmoker | 219 (73.0) | 5830 (72.1) | 1.00 (reference) | - | 1.00 (reference) | - | |
| <20 | 29 (9.7) | 1204 (14.9) | 0.65 (0.43-0.97) | 0.036 | 0.67 (0.45-1.01) | 0.057 | |
| 20- | 18 (6.0) | 349 (4.3) | 1.32 (0.80-2.19) | 0.282 | 1.37 (0.82-2.30) | 0.225 | |
| 30- | 22 (7.3) | 313 (3.9) | 1.71 (1.07-2.73) | 0.026 | 1.80 (1.14-2.91) | 0.016 | |
| 40- | 12 (4.0) | 392 (4.8) | 0.74 (0.40-1.37) | 0.344 | 0.82 (0.44-1.53) | 0.525 | |
| *P*_trend_ |  |  | 0.009 |  | 0.010 |  | |
| Family smoking (exposure to secondhand smoking) | |  |  |  |  |  | |
| No | 168 (56.0) | 4617 (57.1) | 1.00 (reference) | - | 1.00 (reference) | - | |
| Yes | 132 (44.0) | 3471 (42.9) | 1.03 (0.81-1.31) | 0.791 | 1.01 (0.80-1.29) | 0.928 | |
| Duration of smoking(years) |  |  |  |  |  |  | |
| Nonsmoker | 219 (73.0) | 5830 (72.1) | 1.00 (reference) | - | 1.00 (reference) | - | |
| <20 | 11 (3.7) | 808 (10.0) | 0.38 (0.20-0.70) | 0.002 | 0.39 (0.21-0.73) | 0.003 | |
| 20- | 42 (14.0) | 892 (11.0) | 1.19 (0.84-1.70) | 0.335 | 1.29 (0.89-1.85) | 0.175 | |
| 35- | 25 (8.3) | 498 (6.2) | 1.23 (0.79-1.93) | 0.364 | 1.30 (0.82-2.06) | 0.260 | |
| 50- | 3 (1.0) | 60 (0.7) | 1.23 (0.38-4.00) | 0.373 | 1.38 (0.42-4.54) | 0.600 | |
| *P*_trend_ |  |  | 0.014 |  | 0.011 |  | |
| Age at start of smoking(years) |  |  |  |  |  |  | |
| Nonsmoker | 219 (73.0) | 5830 (72.1) | 1.00 (reference) | - | 1.00 (reference) | - | |
| 25- | 10 (3.3) | 239 (3.0) | 1.04 (0.54-2.01) | 0.916 | 1.10 (0.56-2.17) | 0.778 | |
| 20- | 24 (8.0) | 344 (4.3) | 1.75 (1.11-2.76) | 0.016 | 1.87 (1.17-2.99) | 0.009 | |
| <20 | 47 (15.7) | 1675 (20.7) | 0.74 (0.53-1.04) | 0.081 | 0.78 (0.55-1.10) | 0.147 | |
| *P*_trend_ |  |  | 0.011 |  | 0.011 |  | |
| ^a^ Adjusted for Gender, Age, BMI. | | | | | | |  |
| ^b^ Adjusted for Gender, Age, BMI, Annual income per capita (RMB), Education, alcohol units consumed per day. | | | | | | |  |

| **Table S3.** Sensitivity analysis of ORs (95% CIs) for alcohol-related variables with gastroenteritis, restricted to cases diagnosed using high-reliability methods. | | | | | | |
| --- | --- | --- | --- | --- | --- | --- |
|  | **Cases  (n=300)  n(%)** | **Controls （n=8088）  n(%)** | **Adjusted OR^a^ （95%CI）** | ***P*** | **Adjusted OR^b^（95%CI）** | ***P*** |
| Alcohol units consumed per day |  |  |  |  |  |  |
| Nondrinker | 246 (82.0) | 6686 (82.7) | 1.00 (reference) | - | 1.00 (reference) | - |
| <4 | 11 (3.7) | 272 (3.4) | 1.03 (0.55-1.92) | 0.935 | 0.96 (0.51-1.79) | 0.889 |
| 4- | 26 (8.7) | 716 (8.9) | 0.89 (0.57-1.37) | 0.588 | 0.80 (0.51-1.25) | 0.318 |
| 8- | 17 (5.7) | 414 (5.1) | 1.01 (0.60-1.72) | 0.970 | 0.91 (0.53-1.57) | 0.741 |
| *P*_trend_ |  |  | 0.954 |  | 0.798 |  |
| Duration of drinking (years) |  |  |  |  |  |  |
| Nondrinker | 246 (82.0) | 6686 (82.7) | 1.00 (reference) | - | 1.00 (reference) | - |
| <20 | 12 (4.0) | 478 (5.9) | 0.66 (0.36-1.19) | 0.166 | 0.63 (0.34-1.14) | 0.127 |
| 20- | 31 (10.3) | 638 (7.9) | 1.20 (0.79-1.82) | 0.389 | 1.07 (0.70-1.64) | 0.753 |
| 35- | 11 (3.7) | 286 (3.5) | 0.90 (0.47-1.72) | 0.754 | 0.79 (0.41-1.52) | 0.473 |
| *P*_trend_ |  |  | 0.364 |  | 0.377 |  |
| Age at starting drinking (years) |  |  |  |  |  |  |
| Nondrinker | 246 (82.0) | 6686 (82.7) | 1.00 (reference) | - | 1.00 (reference) | - |
| 25- | 6 (2.0) | 138 (1.7) | 1.03 (0.44-2.40) | 0.948 | 0.90 (0.38-2.12) | 0.808 |
| 20- | 12 (4.0) | 222 (2.7) | 1.33 (0.72-2.47) | 0.366 | 1.16 (0.62-2.17) | 0.652 |
| <20 | 36 (12.0) | 1042 (12.9) | 0.86 (0.59-1.26) | 0.446 | 0.80 (0.54-1.17) | 0.249 |
| *P*_trend_ |  |  | 0.642 |  | 0.611 |  |
| Cumulative amount of drinking (unit-years) | |  |  |  |  |  |
| Nondrinker | 246 (82.0) | 6686 (82.7) | 1.00 (reference) | - | 1.00 (reference) | - |
| <40 | 8 (2.7) | 252 (3.1) | 0.82 (0.40-1.69) | 0.593 | 0.79 (0.38-1.62) | 0.515 |
| 40- | 6 (2.0) | 223 (2.8) | 0.70 (0.30-1.61) | 0.398 | 0.65 (0.28-1.51) | 0.317 |
| 80- | 7 (2.3) | 208 (2.6) | 0.84 (0.38-1.82) | 0.652 | 0.76 (0.35-1.67) | 0.495 |
| 120- | 33 (11.0) | 719 (8.9) | 1.12 (0.74-1.68) | 0.611 | 0.98 (0.64-1.50) | 0.939 |
| *P*_trend_ |  |  | 0.807 |  | 0.782 |  |
| ^a^ Adjusted for Gender, Age, BMI. | | | | | | |
| ^b^ Adjusted for Gender, Age, BMI, Annual income per capita (RMB), Education, Number of cigarettes per day. | | | | | | |

| **Table S4.** Crude ORs of the association between dietary intake frequency and risk of gastroenteritis | | |
| --- | --- | --- |
|  | **Crude OR** | ***P*** |
| **Vegetables and edible fungi** |  |  |
| Coriander | 1.05(0.94-1.17) | 0.391 |
| Chrysanthemum coronarium | 0.91(0.81-1.02) | 0.104 |
| Amaranthustricolor | 0.84(0.72-0.98) | 0.028 |
| Spinach | 0.69(0.60-0.79) | <0.001 |
| Baby bok choy | 1.28(1.22-1.36) | <0.001 |
| Garlic chives | 0.97(0.89-1.07) | 0.551 |
| Choy sum | 0.81(0.74-0.88) | <0.001 |
| Garlic bolt | 0.77(0.64-0.93) | 0.007 |
| Lettuce | 1.14(0.96-1.35) | 0.148 |
| Tomato | 1.20(1.11-1.30) | <0.001 |
| Onion and garlic | 0.93(0.86-1.00) | 0.042 |
| Dried mushroom | 0.83(0.75-0.93) | <0.001 |
| Dried laver | 0.90(0.81-0.99) | 0.029 |
| **Fruits and nuts** |  |  |
| Orange | 0.73(0.66-0.81) | <0.001 |
| Citrus | 0.64(0.57-0.71) | <0.001 |
| Strawberry | 0.75(0.68-0.83) | <0.001 |
| Pineapple | 0.79(0.71-0.88) | <0.001 |
| Banana | 1.15(1.07-1.25) | <0.001 |
| Hawthorn | 0.85(0.76-0.94) | 0.002 |
| Peanut | 1.01(0.92-1.11) | 0.833 |
| Walnut | 0.94(0.84-1.05) | 0.252 |
| **Cereals and bean food** |  |  |
| Corn | 1.16(1.08-1.25) | <0.001 |
| Corn flour | 1.06(1.02-1.10) | 0.002 |
| Soy bean | 1.02(0.96-1.09) | 0.577 |
| mung bean | 0.81(0.73-0.91) | <0.001 |
| Adzuki bean | 0.90(0.84-0.97) | 0.008 |
| Yuba | 0.87(0.79-0.97) | 0.012 |
| Fermented bean curd | 0.86(0.78-0.96) | 0.007 |
| **Livers** |  |  |
| Pork liver | 0.63(0.56-0.71) | <0.001 |
| Sheep liver | 0.81(0.73-0.90) | <0.001 |
| Chicken liver | 0.90(0.81-1.00) | 0.057 |
| Duck liver | 0.86(0.77-0.96) | 0.007 |
| **Pickled food** |  |  |
| Pickled radish | 1.00(0.95-1.04) | 0.835 |
| Pickled cucumber | 0.67(0.61-0.72) | <0.001 |
| Pickled mustard tuber | 0.84(0.79-0.89) | <0.001 |
| Pickled root mustard | 0.89(0.83-0.95) | <0.001 |
| Pickled potherb mustard | 1.14(1.05-1.24) | 0.002 |
| Salted meat | 0.80(0.73-0.87) | <0.001 |
| Salted fish | 1.30(1.20-1.41) | <0.001 |
| Salted duck egg | 0.72(0.67-0.78) | <0.001 |
| **Fried food** | 1.09(1.02-1.16) | 0.012 |
| **Yeast products** | 1.06(0.97-1.15) | 0.227 |

**
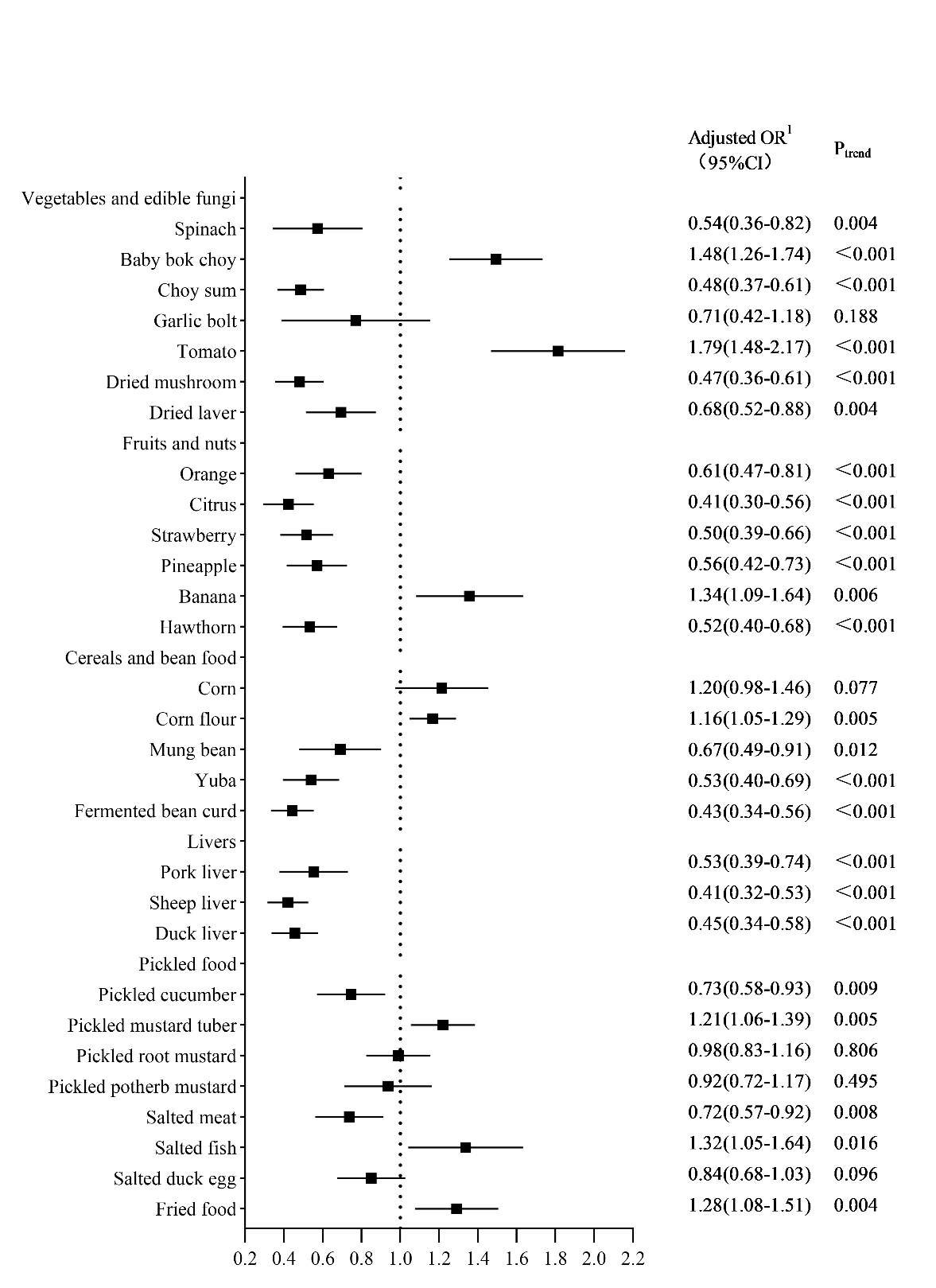
**

**Figure S1**. Association between dietary intake frequency and risk of gastroenteritis in the sensitivity analysis restricted to cases with high-reliability diagnosis.

Note: **^1^**Adjusted for gender, age, BMI, annual income per capita (RMB), education, number of cigarettes per day, alcohol units consumed per day.
